# Alarm-Related Workload in Default and Modified Alarm Settings and the Relationship Between Alarm Workload, Alarm Response Rate, and Care Provider Experience: Quantification and Comparison Study

**DOI:** 10.2196/11704

**Published:** 2018-10-23

**Authors:** Manikantan Shanmugham, Lesley Strawderman, Kari Babski-Reeves, Linkan Bian

**Affiliations:** 1 Department of Industrial and Systems Engineering Mississippi State University Mississippi State, MS United States

**Keywords:** clinical alarms, fatigue, physiologic monitoring, nursing, workload

## Abstract

**Background:**

Delayed or no response to impending patient safety–related calls, poor care provider experience, low job satisfaction, and adverse events are all unwanted outcomes of alarm fatigue. Nurses often cite increases in alarm-related workload as a reason for alarm fatigue, which is a major contributor to the aforementioned unwanted outcomes. Increased workload affects both the care provider and the patient. No studies to date have evaluated the workload while caring for patients and managing alarms simultaneously and related it to the primary measures of alarm fatigue—alarm response rate and care provider experience. Many studies have assessed the effect of modifying the default alarm setting; however, studies on the perceived workload under different alarm settings are limited.

**Objective:**

This study aimed to assess nurses’ or assistants’ perceived workload index of providing care under different clinical alarm settings and establish the relationship between perceived workload, alarm response rate, and care provider experience.

**Methods:**

In a clinical simulator, 30 participants responded to alarms that occurred on a physiological monitor under 2 conditions (default and modified) for a given clinical condition. Participants completed a National Aeronautics and Space Administration-Task Load Index questionnaire and rated the demand experienced on a 20-point visual analog scale with *low* and *high* ratings. A correlational analysis was performed to assess the relationships between the perceived workload score, alarm response rate, and care provider experience.

**Results:**

Participants experienced lower workloads when the clinical alarm threshold limits were modified according to patients’ clinical conditions. The workload index was higher for the default alarm setting (57.60 [SD 2.59]) than for the modified alarm setting (52.39 [SD 2.29]), with a statistically significant difference of 5.21 (95% CI 3.38-7.04), *t*_28_=5.838, *P*<.05. Significant correlations were found between the workload index and alarm response rate. There was a strong negative correlation between alarm response rate and perceived workload, *ρ*_28_=−.54, *P*<.001 with workload explaining 29% of the variation in alarm response rate. There was a moderate negative correlation between the experience reported during patient care and the perceived workload, *ρ*_28_=−.49, *P*<.05.

**Conclusions:**

The perceived workload index was comparatively lower with alarm settings modified for individual patient care than in an unmodified default clinical alarm setting. These findings demonstrate that the modification of clinical alarm limits positively affects the number of alarms accurately addressed, care providers’ experience, and overall satisfaction. The findings support the removal of nonessential alarms based on patient conditions, which can help care providers address the remaining alarms accurately and provide better patient care.

## Introduction

### Background

Physiological monitor alarms and alerts specifically designed by medical device manufacturers are intended to alert clinicians to any deviation of physiological signals from the normal value. Although these devices ensure that doctors and nurses are always informed of physiological changes so as to respond to important deterioration events quickly, they generate very frequent alarms, of which a significant proportion are false [1-5]. Most of these alarms are not relevant to making clinical decisions, providing patient care, or ensuring patients’ safety. About 70% of the alarms occurring in adult intensive care units do not add any value to the nurses’ work process when monitoring patients [6].

Clinical alarms have received immense attention from clinicians, hospital administrators, and watchdog agencies, especially after the US Food and Drug Administration reported 566 alarm-related patient deaths [7,8]. The task of separating the true, actionable alarms from the false or nonactionable alarms lies with the clinicians responsible for responding to the alarms, who in most settings are nurses and their assistants. Alarm fatigue among health care workers, especially nurses, poses a risk to patient safety [9,10]. Upon deciding and initiating appropriate medical treatment, doctors hand off patients from their care to nurses and their assistants during recovery. Patients need to be continuously monitored during this recovery phase for any status changes [11]. When caring for multiple patients, nurses are exposed to numerous alarms per patient per shift and over time become fatigued due to an overwhelming number of alarms [12]. A frequently suggested solution to reduce fatigue is to adjust alarm parameters to suit patient conditions or a standard hospital protocol rather than using textbook normal values or default settings. However, the outcome of this suggestion was mixed [13,14].

Several types of devices—infusion pumps, physiological monitors, and therapy delivery devices—are used in typical patient care settings, and multiple alarms from these devices can cause information overload, leading to clinical errors and poor overall patient outcomes. During clinical alarm management, nurses perform many activities that require excessive cognitive processing, which may contribute to sensory overload, and therefore, their alertness may decrease and errors may occur [15]. Particularly, mental overload may decrease the functioning of working memory. Therefore, assessing the mental workload of attending nurses while they operate these medical devices and monitor patients using physiological monitors is important. Although fatigue and workload are conceptually different, they are closely related. Some researchers have described alarm fatigue as a multicausal, multidimensional, nonspecific, and subjective phenomenon resulting from prolonged activity and psychological, socioeconomic, and environmental factors that affect both the mind and the body [16]. Therefore, assessing mental workload during alarm management will help understand alarm fatigue better. Nurses are an important resource who directly affect the health care system; therefore, ensuring optimal workload level is imperative [17].

### Objective

Although several studies have reported that nurses’ fatigue contributes to alarm mismanagement, no studies have quantified fatigue during alarm management and its effect on patient care quality and outcome. Little research has investigated workload and its correlation with alarm hazards and nurse response time. Given that clinical alarm management is a complex area in its infancy, cognitive workload cannot be described using 1 dimension or characteristic. A multidimensional scale is needed to quantify the mental workload. The National Aeronautics and Space Administration Task Load Index (NASA-TLX) provides a subjective measure of mental demand, physical demand, and temporal demand along with subjects’ own performance, effort, and frustration [18]. Overall workload is measured by summing the scores on the 6 subscales. Although some studies have assessed mental workload in a clinical setting, the specific impact of increased workload on alarm management, response rate, and error rate has not been examined [19-21]. In subjective mental workload, the worker knows the amount of work needed to meet a particular demand. Subjective workload scales have been a familiar part of the human factors and ergonomics tool kit since the 1980s [22]. This study aimed to assess whether any changes in situational complexity, which is differentiated alarm settings, influence the subjective and physiological levels of mental workload and affect the care provider’s experience while caring for patients.

## Methods

### Design, Sample, and Setting

The Mississippi State University’s institutional review board approved this study, and participants’ implied consent was obtained. This study was conducted in a clinical simulator. A total of 30 participants (23 females and 7 males) aged 24 to 60 years (mean 40.66 [SD 9.85]) were recruited. Participants were recruited from hospitals in the Pacific Northwest area of the western United States by word of mouth, phone calls, and flyer postings. Demographic data are presented in Table 1. Participants were randomly assigned to 1 of the 2 alarm threshold groups, default alarm setting and modified setting. Inclusion criteria for the study were medical alarm exposure and basic patient care experience. There were no exclusion criteria. The entire experiment was conducted in 2 waves over the course of 2 weeks. A week was allocated for each alarm setting—default alarm threshold and modified setting. The clinical simulator is equipped with modern physiological monitors and with intensive care equipment for life support, such as infusion and syringe pumps. The simulator setup for experiments was a progressive step-down care unit (patients in this unit are typically low-risk and in the recovery phase of their clinical condition). The entire session was observed through a one-way mirror in the simulator, and data were recorded.

**Table 1:** Demographic data.

Variables		Statistics
Age in years, mean (SD)		40.6 (9.9)
**Gender, n (%)**		
	Female	23 (77)
	Male	7 (23)
**Nursing background, n (%)**		
	Registered nurse	10 (33)
	Nurse assistants (CNAs^a^)	20 (67)
**Years of experience in managing device alarms, n (%)**		
	None	0
	Less than 1 year	1 (3)
	1-3 years	3 (10)
	3-5 years	9 (30)
	More than 5 years	17 (57)
**Trained on medical device alarms, n (%)**		
	Yes	10 (33)
	No	20 (67)
**Training provided by your institution is adequate, n (%)^b^**		
	Yes	5 (17)
	No	14 (47)
**Did your assigned unit provide any training? n (%)^b^**		
	Yes	7 (23)
	No	8 (27)
**Educational background, n (%)**		
	CNAs^a^ or other	20 (67)
	Associates	4 (13)
	Bachelors	4 (13)
	Graduate and more	2 (7)
**Any other certifications? n (%)^b^**		
	Yes	5 (17)
	No	6 (20)

^a^CNA: certified nursing assistant.

^b^Percentage does not equal 100 due to missing responses.

### Procedure, Instrumentation, and Data Collection

The patient condition to be monitored was kept constant to reduce variability. As previous studies have shown that a typical nurse in a progressive care unit does not spend their entire time solely on alarm management and performs other duties for up to 3 patients [23-25], a similar set up was reproduced in a clinical simulator for this experiment. In total, 3 male patient mannequins (SimMan), identified as M-1, M-2, and M-3, and considered low risk based on the Goldman risk chart, were placed in supine positions. M-1 was instrumented with a ProSim SpotLight pulse oximeter simulator (Fluke Bio, Bothell, WA). A physiological monitor (Nellcor with software algorithm *Smart SatSec* feature for customization) connected to the pulse oximeter simulator presented the alarms shown in Table 2. The physiological monitor was set at default for the default setting portion of the experiment, and Smart SatSec was used for the modified setting. Alarms (shown in Table 2) were presented on the screen at a programmed time interval using auto sequence mode. For both settings, the software algorithm was programmed to keep the alarm available for 75 seconds and automatically stop when the time lapsed. M-2 and M-3 did not require monitoring; they were simply recovering from minor outpatient surgical procedures. These mannequins were included to emulate a progressive care unit as closely as possible. Participants performed other assigned *dummy* patient-care tasks on these mannequins as part of the experiment. The additional tasks are described in the following section. Participants were strongly encouraged to complete all dummy tasks. These tasks were also set at the same difficulty level between different alarm conditions (normal alarm threshold and modified setting) to minimize variability. No experimental data other than completion rates were recorded on these tasks. The independent variables were the 2 alarm settings, and the dependent variables were alarm response rate, care provider experience, and overall satisfaction. After providing their background and demographic information, participants rated their care provider experience and overall satisfaction on a 5-point Likert scale survey. Furthermore, the percentage of incorrectly addressed alarms out of the total number of addressed alarms, defined as the error rate, was computed and used as dependent variable.

### Various Alarms

All types of alarms allowed by the physiologic monitor manufacturer were considered in this study. They are defined as follows. An actionable alarm is an alarm that requires a clinician's intervention or warrants a clinician's input or interaction with other clinicians or patients. This alarm should lead to immediate intervention, but due to alarm fatigue could go unwitnessed or misinterpreted by the attending clinician. Actionable alarms require timely intervention to prevent an adverse event. A nonactionable alarm correctly identifies the underlying patient's physiologic condition, but does not require intervention. Its validity is based on waveform quality and accuracy, strength of signals from leads and detectors, and artifact conditions. Transient low-oxygen saturation and heart rate alarms are a few examples of nonactionable alarms. System messages are notifications about medical devices or monitor condition and do not require clinical intervention. A notification about upcoming preventive maintenance of a device is an example for this category. Advisory alarms are status indicators about the parameters monitored and are nonactionable. Elapsed therapy time and amount of remaining fluids left to be delivered are examples for advisory alarms.

**Table 2:** Alarm sequence.

Serial no.	Default setting of the alarm (as released to the hospital floor); total number of alarms=18		Modified to patient condition using *Smart SatSec*; total number of alarms=11	
	Alarm type	Intervention type	Alarm type	Intervention type
1	Advisory	Nonactionable	Removed^a^	Removed^a^
2	Warning	Actionable	Warning	Actionable
3	System message	Nonactionable	Removed^a^	Removed^a^
4	Actionable	Actionable	Actionable	Actionable
5	Warning	Actionable	Warning	Actionable
6	System message	Nonactionable	Removed^a^	Removed^a^
7	Warning	Actionable	Warning	Actionable
8	Actionable	Actionable	Actionable	Actionable
9	Warning	Actionable	Warning	Actionable
10	System message	Nonactionable	System message	Nonactionable
11	System message	Nonactionable	Removed^a^	Removed^a^
12	Advisory	Nonactionable	Removed^a^	Removed^a^
13	Warning	Actionable	Warning	Actionable
14	Advisory	Nonactionable	Advisory	Nonactionable
15	Actionable	Actionable	Actionable	Actionable
16	System message	Nonactionable	System message	Nonactionable
17	Advisory	Nonactionable	Removed^a^	Removed^a^
18	Advisory	Nonactionable	Removed^a^	Removed^a^

^a^These alarms were not presented. Removed alarms: 5 premature ventricular contraction, 1 missed beat, and 1 noninvasive blood pressure.

#### Additional Task Details

The calls were made through an intercom system from outside the simulator, and participants were prompted using the simulator voice communication system at the appropriate time to make calls. Completion rates of tasks in this session were recorded but were not analyzed. Participants were reminded through the microphone when the task was due for completion. To minimize order and interference effects, a 15-min *warm-up* period before starting the session and a 2-min *cooling* period between tasks were provided to participants. During the warm-up period, we discussed alarms and scenarios and asked them to respond verbally. As interference effects between tasks may impact participants’ alarm management, tasks 1 to 4 were presented with a 2-min cooling period before and after:

1. Task 1: call Pharmacy and check the status of ordered medicine for patient mannequin #2 (timing: 2 min into the experiment; call duration: 30 seconds)
2. Task 2: enter blood work result in Epic hospital system software for patient mannequin #3 (timing: 10 min into the experiment; task duration: 2 min)
3. Task 3: administer a bolus dose of pain medicine for patient mannequin #2 (timing: 14 min into the experiment; task duration: 1 min)
4. Task 4: take a call from another hospital unit to receive a patient into this unit (timing: 19 min into the experiment; task duration: 2 min).

#### Data Analysis

Participant characteristics, number of alarms addressed, errors made during management, care provider experience, and overall satisfaction were described using descriptive statistics. To determine any significant differences between the mean alarm response and error rates, 2 one-way analysis of variances (ANOVAs) were performed. As the normality assumptions of the ANOVA were violated according to the Ryan-Joiner method, the Welch-ANOVA method was performed to test hypotheses. A Wilcoxon median rank within-subject test was used to identify any differences in care provider experience and participants’ satisfaction levels when managing alarms in 2 different settings. Relationships between alarm workload and alarm response rate, error rate, care provider experience, and overall satisfaction were established using Spearman rank-order or Pearson product-correlation moment. *P*<.05 was considered statistically significant. IBM SPSS Version 25 for Windows was used for all statistical analyses.

## Results

### Participant Characteristics

Descriptive statistics for the dependent variables are shown in Table 3. A series of chi-square comparison tests were performed to examine whether the NASA-TLX subscale scores differed as a function of demographic characteristics (ie, age, gender, years of experience as a nurse, and alarm management experience). No differences were noted across all analyses (*P*>.05).

### Workload Index

**Figure 1:**
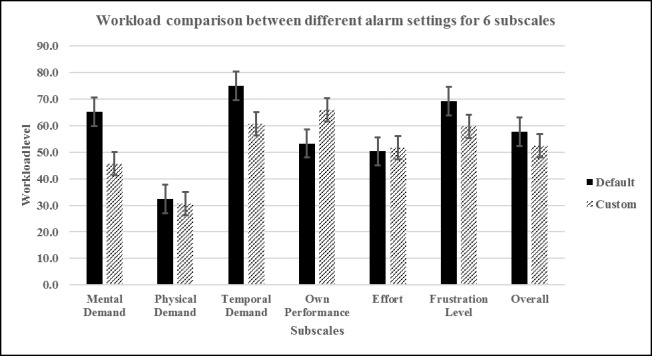
Subscale comparison chart for different alarm settings.

An independent samples *t* test was performed to determine any differences in participants’ perceived workload between modified and default settings. An inspection of a boxplot indicated no outliers in the data. Workload index scores for each of the 6 subscales were normally distributed, as assessed by the Shapiro-Wilks test (*P*>.05), and there was homogeneity of variances, as assessed by Levene test for equality of variances (*P*=.18). The workload index was higher for the default alarm setting (57.60 [SD 2.59]) than for the modified alarm setting (52.39 [SD 2.29]), with a statistically significant difference of 5.21 (95% CI 3.38-7.04), *t*_28_=5.838, *P*<.05. Figure 1 shows participants’ individual ratings on each subscale along with computed overall workload index.

**Table 3:** Descriptive statistics for dependent variables.

Alarm setting and variable		Mean (SD)	Total
**Default**			
	Percentage of alarms addressed	68.9 (10.5)	30
	Error rate	9.5 (6.0)	30
	Care provider experience^a^	2.6 (1.3)	30
	Overall satisfaction^a^	2.5 (0.9)	30
**Modified**			
	Percentage of alarms addressed	86.7 (7.6)	30
	Error rate	2.6 (4.5)	30
	Care provider experience^a^	3.8 (0.8)	30
	Overall satisfaction^a^	4.3 (0.6)	30

^a^Measured on 5-point Likert scale of 1-5 (1=very dissatisfied; 5=very satisfied).

### Alarm Response Rate

A one-way Welch ANOVA was performed to determine whether the alarm response rate was different for the 2 alarm threshold settings. Participants were classified into 2 groups: default (n=15) setting and modified (n=15) setting. Alarm response rate significantly differed between different alarm settings: Welch *F*_1,25.44_=29.05, *P*<.05. Alarm response rate (ie, number of alarms addressed) increased from the default setting to the modified setting due to fewer alarms when physiological monitoring was modified to patient conditions.

#### Relationship Between Alarm Workload and Alarm Response Rate

Pearson product-moment correlation analysis was performed to assess the relationship between workload and the number of alarms addressed (alarm response rate) while providing patient care. The relationship was linear with both variables normally distributed, according to Shapiro-Wilks test (*P*>.05), and there were no outliers. There was a strong negative correlation between alarm response rate and perceived workload, *ρ*_28_=−.54, *P*<.001, with workload explaining 29% of the variation in alarm response rate. The negative correlation indicates that an increase in alarm workload is associated with a reduction in the number of addressed alarms; that is, modification of alarms according to patient conditions in patient-supporting medical devices help reduce care providers’ workload and improve the alarm response rate.

### Error Rate

A one-way Welch ANOVA was performed to determine whether the error rate was different for the default and modified settings. The error rate was significantly different between different alarm settings: Welch *F*_1,25.93_=12.46, *P*<.05. The error rate significantly decreased from the default setting to the modified setting, primarily due to fewer alarms when physiological monitoring was modified to patient conditions.

#### Relationship Between Alarm Workload and Error Rate

A Spearman rank-order correlation analysis was performed to assess the relationship between alarm error rate and perceived workload while providing patient care. A visual inspection of a scatterplot showed a monotonic relationship. There was a strong positive correlation between the number of errors committed (alarm error rate) and the perceived workload, *ρ*_28_=.60, *P*<.05. The number of errors committed by nurses or assistants dropped simultaneously with the corresponding workload, which shows that they are associated with each other in a health care environment.

### Care Provider Experience

A Mann-Whitney U test was performed to determine whether there were differences in care provider experience between default and modified alarm settings. Distributions of care provider ratings for default and modified settings were similar, as assessed by visual inspection. Care provider experience ratings (on a 5-point Likert scale) for the modified setting (mean rank=20.83) were significantly higher than those for the default setting (mean rank=10.17), *U*=32.5, *z*=−3.422, *P*=.001, using an exact sampling distribution for *U*.

#### Relationship Between Alarm Workload and Care Provider Experience

A Spearman rank-order correlation analysis was performed to assess the relationship between perceived workload and care provider experience while providing patient care in a progressive care setting. A visual inspection of a scatterplot showed a monotonic relationship. There was a moderate negative correlation between the experience reported during patient care and the perceived workload, *ρ*_28_=−.49, *P*<.05. The care provider experience, during or after caring for patients, was inversely proportional to the alarm-related workload. It is important to note that the participants were managing alarms along with several patient care tasks to mimic real-world situations. Therefore, any reduction in workload positively impacted care provider experience and well-being at the job.

### Overall Satisfaction

To determine any differences in overall satisfaction between default and modified alarm settings, a Mann-Whitney U test was performed. Distributions of overall satisfaction ratings for default and modified settings were similar, as assessed by visual inspection. Overall satisfaction ratings (on a 5-point Likert scale) for the modified setting (mean rank=21.90) were significantly higher than those for the default setting (mean rank=9.10), *U*=16.5, *z*=−4.146, *P*=.001, using an exact sampling distribution for *U*.

#### Relationship Between Alarm Workload and Overall Satisfaction

A Spearman rank-order correlation analysis was performed to assess the relationship between perceived workload and overall satisfaction while providing patient care in a progressive care setting. A visual inspection of a scatterplot showed a monotonic relationship. There was a strong negative correlation between the overall reported satisfaction and perceived workload, *ρ*_28_=−.69, *P*<.05. The negative correlation indicates that the workload increase is associated with overall satisfaction, which decreased significantly. Therefore, hospital administrators and risk managers should consider customizing alarms in patient-supporting medical products, as it is a key factor of care providers’ satisfaction.

## Discussion

### Principal Findings

Delayed or no response to impending patient safety–related calls, poor care provider experience, low job satisfaction, and adverse events are all unwanted outcomes of alarm fatigue. In this study, alteration of alarm limits by customizing the experimental settings based on patients’ conditions resulted in lower NASA-TLX scores than those obtained using the default manufacturer settings. That is, allowing the physiological monitoring device to operate under a default setting based on normal textbook values resulted in more alarms, thereby leading to a higher mental workload while managing these alarms. Higher NASA-TLX scores indicate that alarm management is a complex task and has the potential to induce fatigue. Higher mental workload impacts nurses’ attentiveness, increases the risk of slow responses, and can result in poor task accuracy. The number of alarm signals has been reported to reach several hundred per day for some patients in 1 study, thus creating a high alarm burden for nurses [26]. Nurses will be desensitized by such a high alarm burden and may miss, ignore, or disable alarm signals, which might result in adverse events [27].

The scores on NASA-TLX show that temporal demand, mental demand (MD), and frustration level are the major contributors to alarm workload. This is not surprising, as responding to alarms is secondary to primary care provider tasks such as medication administration, patient assessments, and note updates. In such dual-task systems, time spent on responding to alarms distracts from the primary tasks, and nurses feel pressed for time and frustrated. The higher MD score is attributable to the process involved in analyzing and isolating the source of the alarm, which often requires higher cognitive amplitude.

Participants’ self-reported performance was higher in the modified setting than in the default setting. The higher alarm response rate in the modified setting supports this score. Better alarm response rate is also manifested across 2 other subscales, lower frustration and overall workload index, as shown in Figure 1. Not surprisingly, the subscale scores for physical demand and effort in the modified and default settings were statistically similar and lower compared with other subscales in their respective groups. Although only 4 of the 30 (13%, 4/30) participants provided narrative data, making it difficult to generalize for the entire group, the common theme for the default setting was the excessive number of alarms and tasks. The most important finding is that the number of alarms addressed was inversely proportional to the workload encountered during patient care. Participants were able to address almost all presented alarms when the alarm settings were modified according to patient conditions. This finding is consistent with those of similar alarm setting modification studies, which showed that a 43% reduction in alarms is possible through alarm setting customization [26,27]. Participants also expressed positive views of alarm customization. Some researchers have reported reducing the total number of alarms from 180 per patient per day to 40 through a unit-level standardization project, which included a daily individualization of alarm parameters [28]. More than a 50% reduction in the total rate of alarms per bed per day and a significant decrease in noise are possible by eliminating 3 types of ventricular contraction alarms [29].

Another unique finding of this study is that the alarm workload was directly proportional to the number of errors committed. The decrease in the number of errors is associated with the number of alarms that needed to be addressed during patient care. This suggests that the removal of certain nonessential alarms enabled the nurses to address the remaining important alarms accurately without any or with only minimal errors. The overwhelming number of alarms in the default setting put time pressure on nurses, and thus, they attempted to address more alarms within the limited time and made errors along the way. This can also be seen in a different way—if the number of opportunities (alarms) to make an error is limited, the number of errors committed will likely reduce.

Care provider experience and overall satisfaction were inversely correlated to alarm-related workload. As the alarm-related workload increases—which is typical when the alarms are set at the manufacturer’s default setting—the quality of the experience of care providers caring for patients decreases. When the number of alarms to be assessed and addressed is low or lower they have more time to focus on patient care tasks and carry out other critical administrative tasks. The lesser the job stress and feeling of *burn out*, the higher the job satisfaction and general well-being in a typical health care setting [30]. It is likely that the lesser number of alarms in the modified setting allowed participants to complete all tasks with less time pressure and to be engaged with the system, which was reflected in higher satisfaction scores. The only difference between the default and modified experimental set-up(s) was the total number of alarms. Therefore, changes observed in care provider experience and overall satisfaction were most likely associated with modifications in alarm-related workload. A larger sample population and other types of monitoring devices are needed to determine whether alarm workload is the causal factor.

### Limitations

The entire experiment was executed in a simulator lab setting, which is controlled and supported; therefore, the applicability of the findings should be examined further and may need to be repeated before being implemented into policies and procedures. Future studies should also include additional populations such as physicians, medical assistants, and other therapists who are also part of the patient care team. The sample population was entirely based out of 3 local hospitals in the Pacific Northwest region of the United States. It is well known that the health care field has regional cultures. Future studies should recruit participants across the country and investigate whether the effect of alarm modifications will bring similar benefits under other patient care settings such as intensive care, coronary care, emergency wards, and medical-surgical units.

### Conclusions

The findings of this study show that removal of certain nonessential alarms based on patient condition can result in better care provider experience, reduced mental workload, and higher overall satisfaction. The number of managed alarms is directly proportional to workload and the number of errors (error rate) committed and inversely proportional to alarm response rate and care provider experience. Evidence for optimal alarm settings for physiological monitors and cardiac devices is abundant. Hospital administrators should make efforts to develop appropriate threshold levels for various physiological measures that clinicians monitor for typical patient conditions. This will help reduce the alarm burden for nurses and their aides significantly.

## Abbreviations

ANOVA: analysis of variance
MD: mental demand
NASA-TLX: National Aeronautics and Space Administration-Task Load Index
